# Phosphorylation of TabZIP27 by TaCIPK16 Regulates *TaMAN7-4B* Expression During Cadmium Response in Wheat

**DOI:** 10.3390/plants15203214

**Published:** 2026-10-20

**Authors:** Liwen Wang, Dayong Cui, Yonglan Chang, Fei Zhang, Changliang Du, Rugang Wu, Caibo Zhang, Wenya Li, Wenqian Liu, Yongchao Gong, Yanyan Pu, Junke Zhu

**Affiliations:** 1 School of Life Sciences, Qilu Normal University, Jinan 250200, China; 2 Dezhou Academy of Agricultural Sciences, Dezhou 253015, China; 3 Institute of Crop Germplasm Resources, Shandong Academy of Agricultural Sciences, Jinan 250100, China

**Keywords:** wheat, cadmium, phosphorylation, transcript regulation

## Abstract

Cadmium (Cd) contamination poses a significant threat to wheat productivity and food safety. This study demonstrates that *TaMAN7-4B*, which encodes an endo-β-mannanase involved in the metabolism of cell wall mannans, is significantly upregulated in response to Cd stress, thereby enhancing Cd tolerance by increasing the root cell wall mannan content and facilitating Cd sequestration. Furthermore, we identified the transcription factor TabZIP27 as a direct regulator of *TaMAN7-4B* expression. TabZIP27 is phosphorylated by the kinase TaCIPK16, which stabilizes TabZIP27 by suppressing its proteasome-dependent degradation. This phosphorylation event subsequently promotes the transcription of *TaMAN7-4B*, resulting in enhanced Cd binding within the root cell walls and reduced translocation of Cd from roots to shoots. Our findings elucidate a novel regulatory module, TaCIPK16-TabZIP27-*TaMAN7-4B*, which governs Cd accumulation in wheat, thereby providing potential targets for the breeding of low-Cd cultivars.

## 1. Introduction

Cadmium (Cd) is a pervasive heavy metal commonly found in soil, posing significant threats to both crop growth and human health as it enters the food chain [1]. High concentrations of Cd exposure severely impact various aspects of plant physiology and biology [2,3]. For example, Cd-induced damage to chloroplast structures impedes the accumulation of photosynthetic pigments and reduces photosynthetic rates, thereby affecting crop quality and yield [4,5]. Additionally, Cd toxicity disrupts ion homeostasis in cells and induces the production of reactive oxygen species, leading to metabolic dysregulation [6,7,8]. As a vital food crop, wheat is particularly susceptible to Cd contamination, threatening not only its yield and quality but also posing risks to human and animal health. Consequently, reducing Cd accumulation in wheat is a crucial strategy for mitigating these risks.

The plant cell wall functions as the primary site for the interception of Cd ions prior to their entry into the cell interior. Research indicates that within plant roots, the cell wall serves as the principal accumulation site for Cd [9,10]. This immobilization significantly reduces the translocation of Cd to shoot tissues of the plant, thereby mitigating its toxicity [11]. The Cd-binding capacity of cell walls is largely attributed to their polysaccharide components, including pectin, hemicellulose, cellulose, and mannan [12,13,14]. These components are rich in functional groups, such as hydroxyl (-OH) and carboxyl (-COOH), which facilitate the binding or adsorption of Cd [15]. Research indicates that hemicellulose in root cell walls enhances Cd binding and reduces its transfer to shoots [16]. Hemicellulose can be categorized into four types: xyloglucan, xylan, mannan, and mixed-linkage β-glucan [17]. Mannan is a vital component of hemicellulose, whose structure, content, and modification status play critical and varied roles in plant responses to diverse abiotic stresses, including drought, salinity, and heavy metals [13,18,19,20]. In addition to serving as a structural element that maintains cell wall integrity, mannan functions as a dynamic regulatory molecule that enhances plant adaptability to stress by influencing the mechanical properties of the cell wall, water retention capacity, and signal transduction pathways [21]. In plants, endo-β-mannanase (MAN) is a key enzyme that modifies mannans by randomly hydrolyzing internal β-1,4-D-mannopyranosyl linkages within the mannan backbone [20]. Previous studies have confirmed that *Arabidopsis* MAN responds to Cd stress. For instance, overexpression of *MAN3* results in increased Cd accumulation, while man3 mutants display heightened sensitivity to Cd [22]. Similarly, MAN7 enhances Cd tolerance by modulating the ability of *Arabidopsis* root cell walls to bind Cd [13]. Because the enzymes that build and remodel cell wall mannan, and the basic mechanism by which cell wall polysaccharides bind Cd, are widely conserved among angiosperms, wheat orthologs of *Arabidopsis MAN* genes would be expected to play a comparable role in the root cell wall. However, no *MAN* gene had been functionally characterized in wheat, and it remained unknown whether wheat *MAN* genes contribute to Cd tolerance and which transcription factors control their expression.

The basic leucine zipper (bZIP) transcription factors (TFs) constitute one of the most conserved and functionally diverse TF families in plants and participate in abiotic and biotic stress responses, hormone signaling, and development [23]. bZIP TFs contain a conserved basic region responsible for DNA binding and a leucine zipper involved in dimerization, enabling them to recognize ACGT-containing cis-elements such as the ABA-responsive element (ABRE) [24]. Several bZIP TFs have been implicated in heavy metal responses. For example, *CibZIP29* responds to Cd and enhances Cd tolerance in transgenic *Arabidopsis* [25], whereas *bZIP44* improves Pb tolerance by activating PDR12 in *Arabidopsis* [26]. Notably, bZIP44 also directly regulates *MAN7* expression [13]. The activity of bZIP TFs can also be modulated by upstream kinases and hormone signaling pathways. OsbZIP42, activated by SAPK4, and OsbZIP20, phosphorylated by SAPK10, participate in drought and salt responses [27,28], whereas ZmbZIP76 enhances reactive oxygen species scavenging and ion homeostasis under salt stress [29]. Because Cd stress shares Ca^2+^, reactive oxygen species, and ABA signaling components with drought and salt stress, kinase-mediated phosphorylation of bZIP TFs may represent an important mechanism integrating Cd-responsive signaling.

The CBL-interacting protein kinase (CIPK) family transduces Ca^2+^ signals through phosphorylation cascades that target downstream TFs [30]. Under drought, salinity, or pathogen attack, the CBL-CIPK complex senses cytosolic Ca^2+^ fluctuations and phosphorylates downstream substrates, including TFs, to reprogram stress-responsive gene expression [31,32]. For example, *MdCIPK22* phosphorylates *MdAREB2* to regulate ABA responses in apple [33], and a CIPK1-regulated NAC TF coordinates shoot branching and nitrate accumulation in tomato [34]. CIPKs are therefore attractive upstream regulators of metal-stress bZIP TFs; however, a CIPK-bZIP-cell wall module has not been described in wheat.

Taken together, we hypothesized that a Cd-responsive wheat *MAN* gene remodels root cell wall mannan to sequester Cd, and that its expression is regulated by a phosphorylation-dependent CIPK-bZIP module. To test this hypothesis, we characterized the Cd-responsive wheat gene *TaMAN7-4B* and investigated its role in Cd tolerance and root-to-shoot Cd translocation using RNAi and overexpression lines. We further examined whether the bZIP TF TabZIP27 directly binds to the *TaMAN7-4B* promoter and activates its transcription, and whether TaCIPK16 interacts with and phosphorylates TabZIP27 to enhance its stability and thereby promote *TaMAN7-4B* expression. Our findings establish a TaCIPK16–TabZIP27–*TaMAN7-4B* regulatory module involved in Cd accumulation in wheat and provide potential genetic targets for developing wheat cultivars with reduced Cd accumulation.

## 2. Results

### 2.1. TaMAN7-4B Positively Regulates Cd Tolerance in Wheat

**Figure 1:**
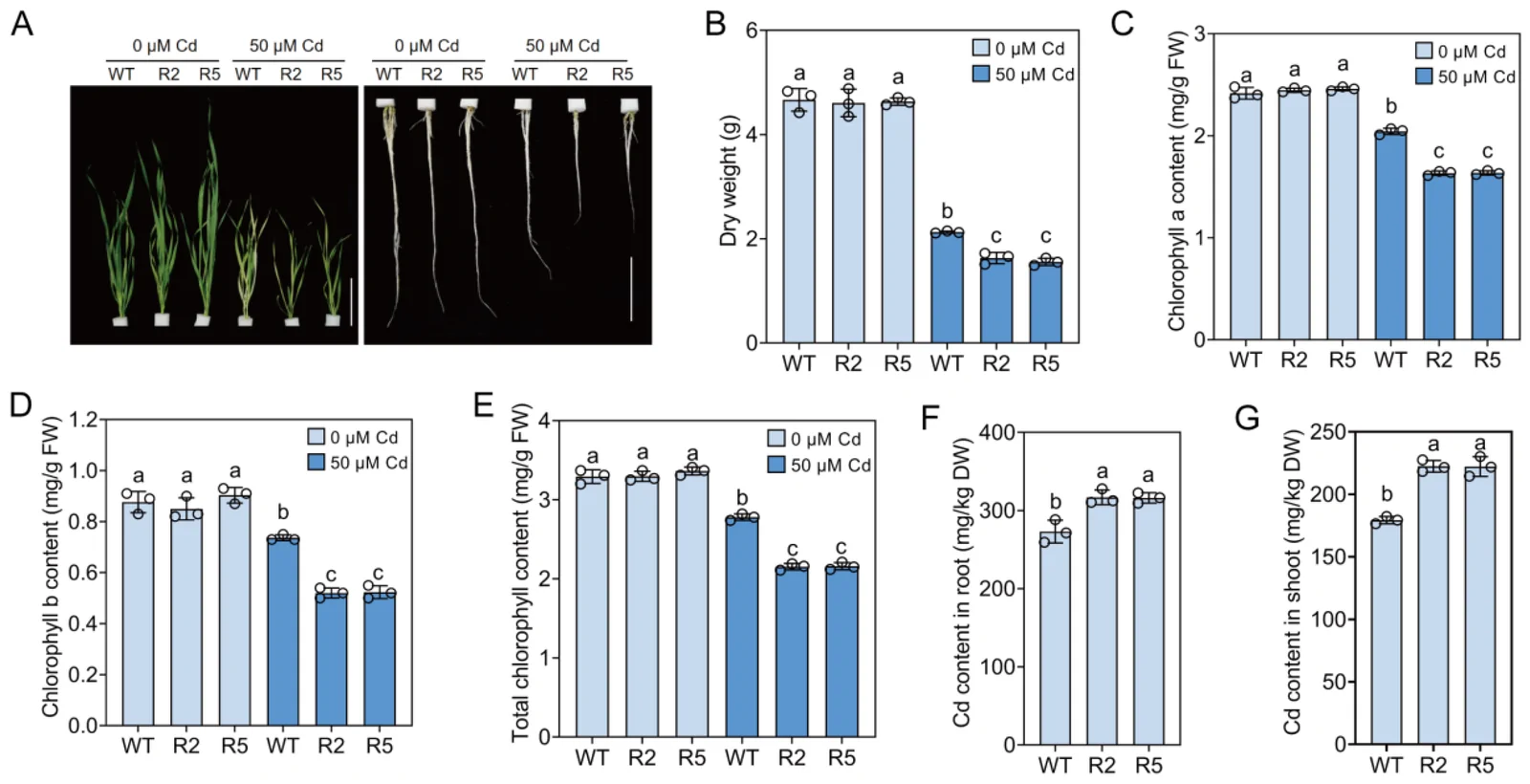
*TaMAN7-4B* silencing reduces Cd tolerance in wheat. (**A**) The phenotypes of WT and *TaMAN7-4B* RNAi wheat seedlings grown with or without 50 μM Cd for 14 d. Scale bar = 5 cm. (**B**) The dry weight, (**C**) chlorophyll a, (**D**) chlorophyll b, and (**E**) total chlorophyll contents, (**F**) Cd content in root, and (**G**) Cd content in shoot of WT and *TaMAN7-4B* RNAi wheat seedlings grown with or without Cd for 14 d. Data are means ± SD (*n* = 3), and different letters above bars indicate significant differences by one-way ANOVA followed by Tukey’s HSD test at *p* < 0.05.

Research has demonstrated that endo-β-mannanase (MAN) plays a significant role in the accumulation of Cd in plants [26]. We identified the *MAN* gene TraesCS4B02G186300 in the transcriptome of wheat following Cd treatment (unpublished data). Sequence alignment revealed that TraesCS4B02G186300 is a homolog of the *Arabidopsis* MAN7 gene, leading to its designation as *TaMAN7-4B*. RT-qPCR analysis demonstrated *TaMAN7-4B* responds to Cd stress in both wheat roots and shoots (Supplementary Figure S1). Subsequently, we constructed wheat plants with downregulated expression of *TaMAN7-4B* using RNA interference (RNAi), with lines R2 and R5 exhibiting the lowest expression levels (Supplementary Figure S2). Analysis of seedling phenotypes indicated no significant differences between wild-type (WT) and *TaMAN7-4B* RNAi wheat when grown in a Cd-free nutrient solution. However, under Cd stress, *TaMAN7-4B* RNAi wheat exhibited lower biomass compared to WT (Figure 1A,B), along with more severe chlorosis and significantly reduced levels of chlorophyll a, chlorophyll b, and total chlorophyll (Figure 1C–E). Cd content analysis revealed that *TaMAN7-4B* RNAi wheat accumulated significantly higher levels of Cd than WT in both root and shoot tissues (Figure 1F,G).

**Figure 2:**
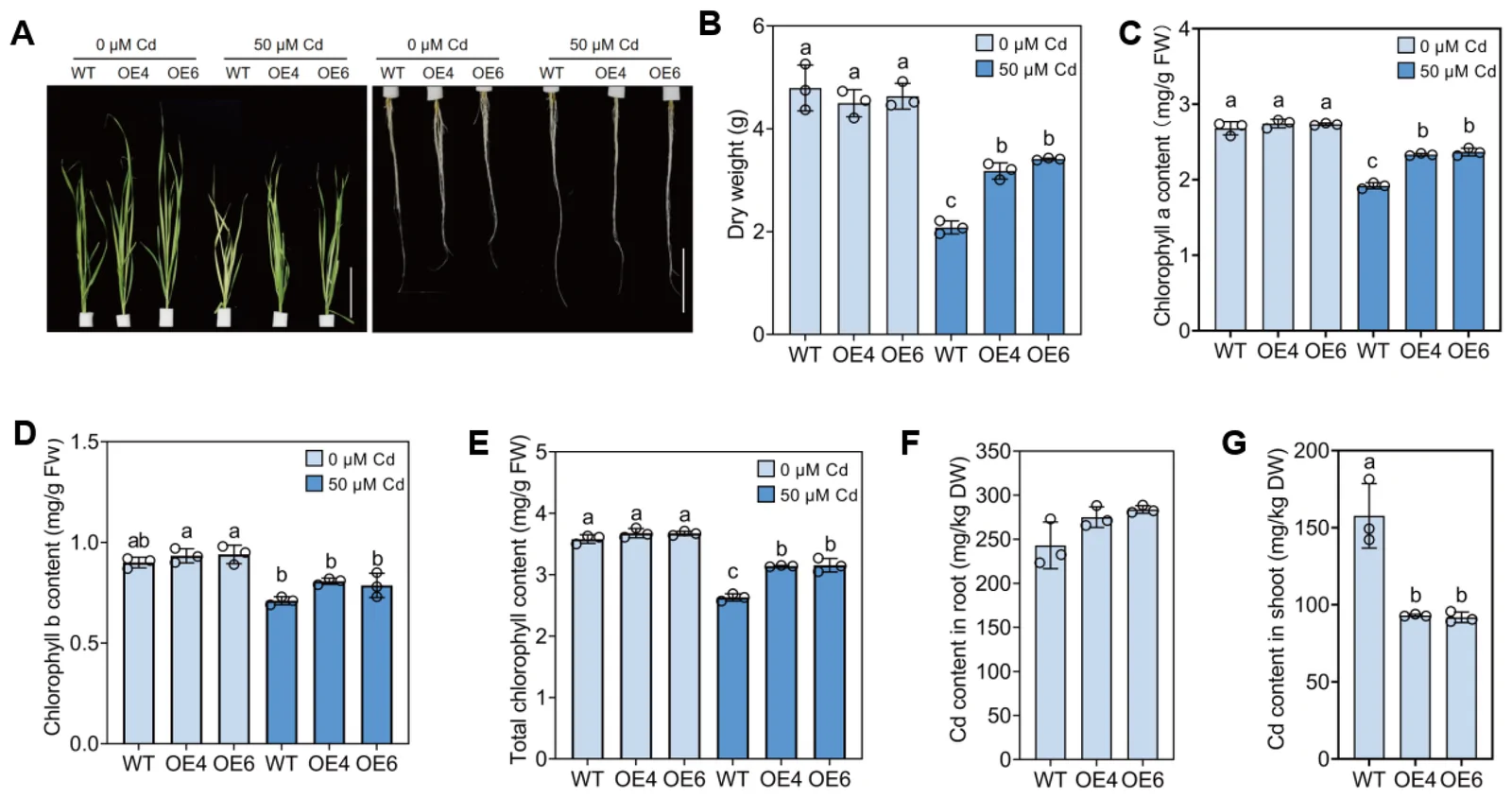
*TaMAN7-4B* overexpression enhances Cd tolerance in wheat. (**A**) The phenotypes of WT and *TaMAN7-4B* overexpression wheat seedlings grown with or without 50 μM Cd for 14 d. Scale bar = 5 cm. (**B**) The dry weight, (**C**) chlorophyll a, (**D**) chlorophyll b, and (**E**) total chlorophyll contents, (**F**) Cd content in root, and (**G**) Cd content in shoot of WT and *TaMAN7-4B* overexpression wheat seedlings grown with or without Cd for 14 d. Data are means ± SD (*n* = 3), and different letters above bars indicate significant differences by one-way ANOVA followed by Tukey’s HSD test at *p* < 0.05.

To investigate the function of *TaMAN7-4B*, we constructed overexpression lines and performed RT-qPCR analysis (Supplementary Figure S3). From this analysis, we selected two overexpression lines, designated as OE4 and OE6. Under Cd stress, both *TaMAN7-4B* overexpression lines demonstrated enhanced Cd tolerance compared to the WT (Figure 2A), as evidenced by improved growth vigor and greater biomass relative to the WT under Cd stress (Figure 2B). Meanwhile, we observed that the overexpression of *TaMAN7-4B* significantly increased the levels of chlorophyll a, chlorophyll b, and total chlorophyll in response to Cd stress (Figure 2C–E). In comparison to the WT, the overexpression of *TaMAN7-4B* did not significantly affect Cd accumulation in the roots (Figure 2F). However, it notably reduced Cd content in the shoots (Figure 2G), indicating that *TaMAN7-4B* overexpression plays a crucial role in reducing Cd translocation from roots to shoots.

Additionally, we compared the Cd translocation factors among WT, *TaMAN7-4B* RNAi, and *TaMAN7-4B* overexpression wheat lines. Our findings indicated that the Cd translocation factor in *TaMAN7-4B* overexpression wheat was significantly lower than that in WT (Supplementary Figure S4). In contrast, no significant difference was observed between *TaMAN7-4B* RNAi and WT, although the mean value was higher in *TaMAN7-4B* RNAi (Supplementary Figure S4). This suggests that *TaMAN7-4B* overexpression inhibits the translocation of Cd to the shoots.

### 2.2. TaMAN7-4B Affects Hemicellulose and Mannose Contents in Wheat Roots

**Figure 3:**
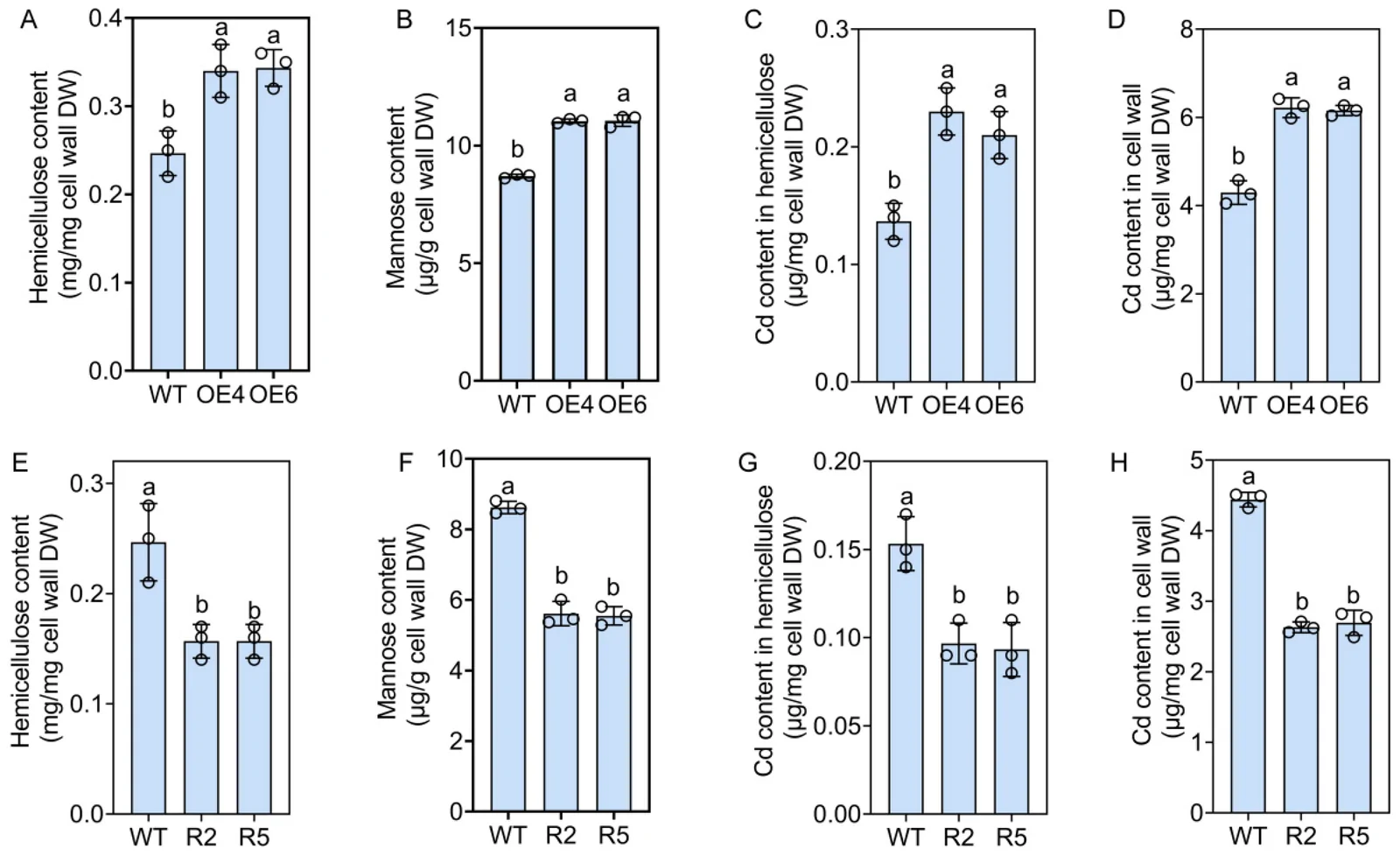
(**A**) Hemicellulose content of WT and *TaMAN7-4B* overexpression wheat. (**B**) Mannose content in WT and *TaMAN7-4B* overexpression wheat. (**C**) Cd content in hemicellulose of WT and *TaMAN7-4B* overexpression wheat. (**D**) Cd content in cell wall WT and *TaMAN7-4B* overexpression wheat. (**E**) Hemicellulose content of WT and *TaMAN7-4B* RNAi wheat. (**F**) Mannose content in WT and *TaMAN7-4B* RNAi wheat. (**G**) Cd content in hemicellulose of WT and *TaMAN7-4B* RNAi wheat. (**H**) Cd content in cell wall of WT and *TaMAN7-4B* RNAi wheat. Data are means ± SD (*n* = 3) and different letters above bars indicate significant differences by one-way ANOVA followed by Tukey’s HSD test at *p* < 0.05.

Previous studies have confirmed that the MAN gene influences the levels of hemicellulose and mannose in the cell walls of *Arabidopsis* [13]. In this study, we investigated whether the expression of *TaMAN7-4B* affects the hemicellulose and mannose content in wheat roots. Our findings demonstrate that the overexpression of *TaMAN7-4B* significantly enhanced the hemicellulose and mannose content in wheat roots (Figure 3A,B). In contrast, RNAi wheat exhibited a markedly lower content of hemicellulose and mannose compared to WT plants (Figure 3E,F). Additionally, we analyzed the Cd content adsorbed by the cell walls and hemicellulose, revealing that the root cell walls and hemicellulose of *TaMAN7-4B* overexpressing wheat absorbed more Cd than those of the WT (Figure 3C,D). Conversely, the Cd content adsorbed by the cell walls and hemicellulose of *TaMAN7-4B* RNAi roots was significantly lower than that of the WT (Figure 3G,H). This suggests that *TaMAN7-4B* overexpression enhances the capacity for Cd adsorption in roots, which explains why the Cd transfer coefficient in *TaMAN7-4B*-overexpressing wheat is significantly lower than that in RNAi wheat.

### 2.3. TabZIP27 Regulates the Expression of TaMAN7-4B

**Figure 4:**
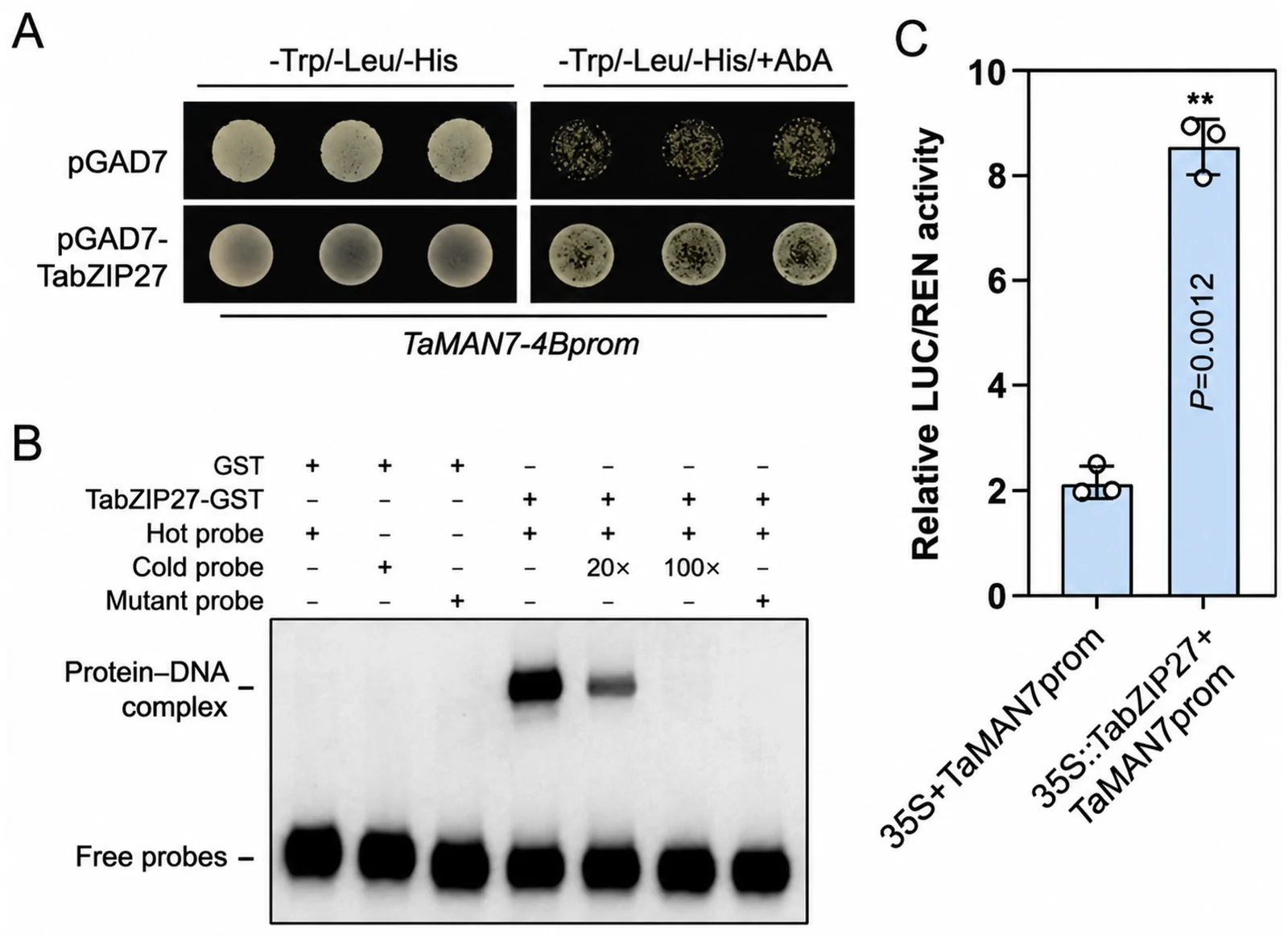
(**A**) Yeast one hybrid assay. A pair of plasmids, pAbAi-*TaMAN7-4Bprom* and pGADT7-*TabZIP27* were introduced into yeast one-hybrid gold and cultured on synthetically defined (SD) medium containing Aureobasidin A (200 ng/mL; AbA200) or not (−AbA) at 30 °C for 5 d. A pair of plasmids pAbAi-*TaMAN7-4Bprom* and pGADT7 were used as the negative control. (**B**) EMSA showing TabZIP27 binding to *TaMAN7-4B* promoter specifically. The sequences of hot probe, cold probe, and mutant probe have been shown in Supplementary Table S1. The ‘+’ and ‘−’ symbols indicate the presence and absence of the indicated components, respectively. (**C**) Relative luciferase (LUC) activity of *TaMAN7-4Bprom*-LUC reporter after co-expression of *35S*::*TabZIP27* in tobacco. Empty vector was used as the effector plasmid control. Error bars represent ± SD (*n* = 3). The asterisks indicate significant differences by a two-tailed Student’s *t*-test at *p* < 0.05.

Using the yeast one-hybrid (Y1H) screening system, we identified TabZIP27 as a potential upstream regulator of the *TaMAN7-4B* gene. To verify whether TabZIP27 can bind to the promoter of *TaMAN7-4B*, we conducted Y1H assays with the full-length TabZIP27 protein and the promoter region of *TaMAN7-4B* (*TaMAN7-4Bprom*), which spans from −1 to −2001 bp upstream of the start codon. Yeast cells containing the bait vector pAbAi, which harbored the inserted *TaMAN7-4B* promoter, were transformed with the AD-TabZIP27 vector. These transformed cells were capable of growth on -Trp/-Leu/-His/+AbA (Aureobasidin A) medium, indicating that *TaMAN7-4B* is a target of TabZIP27 (Figure 4A). Since bZIP TFs bind to *cis*-elements containing ACGT in the promoters of target genes, we analyzed the promoter sequence of *TaMAN7-4B* and identified a region containing ACGT (−1661 to −1637) (Supplementary Figure S5). Subsequently, we performed EMSA using recombinant GST-TabZIP27 to determine whether this segment is bound by TabZIP27. The results demonstrated that TabZIP27 exhibits binding affinity for the biotin-labeled probe synthesized from this sequence, but fails to bind the mutated probe of this *cis*-element (Figure 4B).

To investigate whether TabZIP27 mediates the transcriptional activation of *TaMAN7-4B*, we conducted a transient dual-luciferase reporter assay. In this assay, the promoter of *TaMAN7-4B* was cloned upstream of the firefly luciferase gene to serve as the reporter vector, while the *35S*:*TabZIP27* construct functioned as the effector vector (Supplementary Figure S6). Compared to the empty effector construct and LUC reporter, tobacco co-transfected with both the reporter and effector vectors demonstrated significantly enhanced luciferase activity (Figure 4C). These results indicate that TabZIP27 positively and directly regulates the transcription of *TaMAN7-4B*.

### 2.4. TaCIPK16 Phosphorylates TabZIP27 Both In Vivo and In Vitro

**Figure 5:**
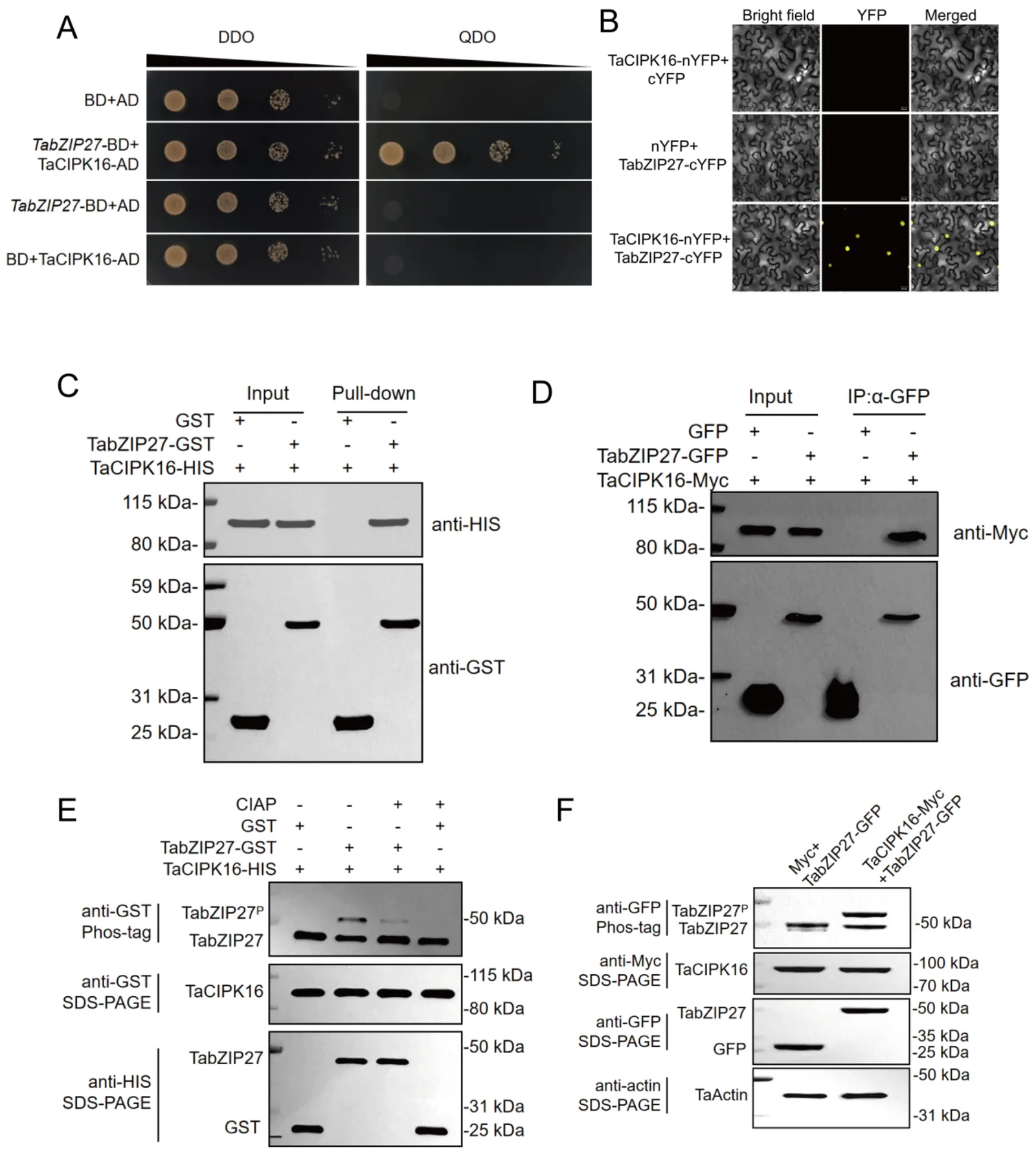
(**A**) Protein interaction between TabZIP27 and TaCIPK16 in an Y2H assay. DDO: SD/-Leu-Trp. QDO: SD/-Ade-His-Leu-Trp. (**B**) BiFC assay showing that TabZIP27 interaction with TaCIPK16 in tobacco. Scale bars = 20 μm. (**C**) GST pull-down assay showing TabZIP27 interaction with TaCIPK16. (**D**) Co-IP analysis showing the interaction TabZIP27 with TaCIPK16. (**E**) In vitro phosphorylation activity of TabZIP27 protein was analyzed using phos-tag acrylamide. The upper band (TabZIP27-P) corresponds to phosphorylated TabZIP27, whereas the lower band corresponds to non-phosphorylated TabZIP27; treatment with calf intestinal alkaline phosphatase (CIAP) shifted the upper band to the lower position, confirming that the retarded migration band of TabZIP27 in the phos-tag gel originated from phosphorylation modification. (**F**) In vivo phosphorylation of TabZIP27 mediated by TaCIPK16 was achieved via *Agrobacterium*-mediated transient expression in tobacco leaves. The ‘+’ and ‘−’ symbols indicate the presence and absence of the indicated proteins or reagents, respectively.

Given that the stability of TFs is frequently regulated by post-translational modifications, we conducted a yeast library screen and identified TaCIPK16 as a potential interacting protein of TabZIP27 (Figure 5A). Subsequently, we investigated the interaction between TabZIP27 and TaCIPK16 in vivo using BiFC assays. When TaCIPK16-nYFP and TabZIP27-cYFP were transiently co-expressed in tobacco leaves, we observed strong yellow fluorescent protein (YFP) signals in the nuclei of epidermal cells (Figure 5B). TabZIP27 was predominantly localized in the nucleus (Supplementary Figure S6A), while TaCIPK16 exhibited significant subcellular localization in both the cytoplasm and nucleus (Supplementary Figure S6B), suggesting an overlap in their subcellular distribution. To determine whether TaCIPK16 could directly interact with TabZIP27 in vitro, we performed a GST-pull down assay. As illustrated in Figure 5C, HIS-TaCIPK16 was successfully pulled down by GST-TabZIP27, but not by GST alone, indicating a direct interaction between TaCIPK16 and TabZIP27 in vitro. Furthermore, we confirmed the interaction between TaCIPK16 and TabZIP27 through Co-IP assays. When HIS-TaCIPK16 was incubated with total protein extracts from TabZIP27-GFP plants, HIS-TaCIPK16 was detected within the immunoprecipitated TabZIP27-GFP complex (Figure 5D). Collectively, these results demonstrate that TabZIP27 physically interacts with TaCIPK16 both in vivo and in vitro.

CIPK has been shown to phosphorylate multiple TFs, thereby influencing their stability. Consequently, we further examined the phosphorylation of TabZIP27 by TaCIPK16. We expressed the recombinant proteins GST-TaCIPK16, HIS-TabZIP27, and GST. The in vitro phosphorylation assays revealed that phosphorylated bands were detected in the anti-HIS blot of HIS-TabZIP27. However, this phosphorylation signal was eliminated in the presence of calf intestinal alkaline phosphatase (CIAP) (Figure 5E). Additionally, we performed transient expression and phosphorylation analysis in tobacco leaves using the constructed GFP-TaCIPK16 and Myc-TabZIP27, which showed the presence of phosphorylated bands (Figure 5F). These findings confirm that TabZIP27 can be phosphorylated by TaCIPK16.

### 2.5. The TaCIPK16-TabZIP27 Module Regulates the Stability of TabZIP27

**Figure 6:**
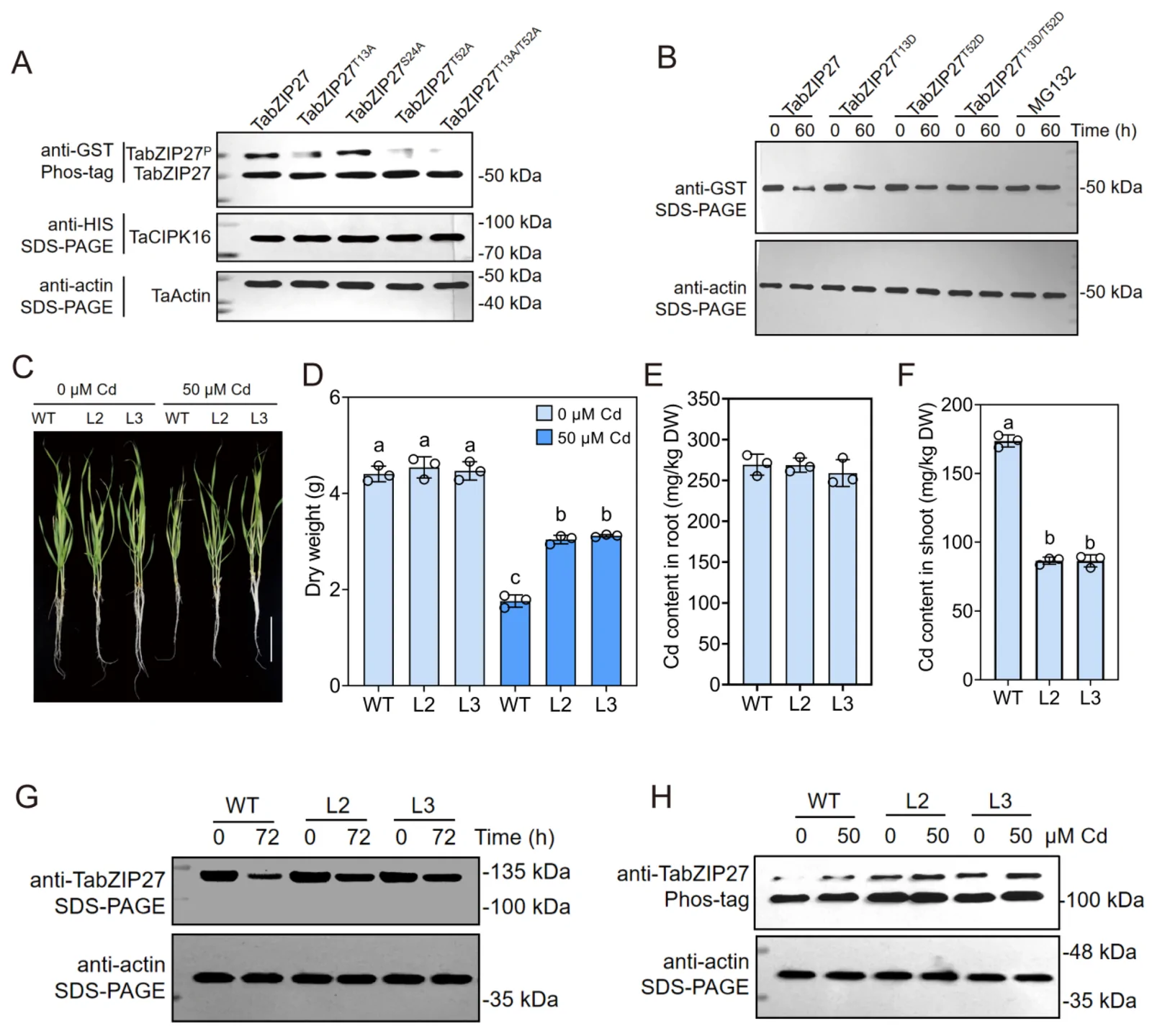
(**A**) In vitro phosphorylation activity of TabZIP27, TabZIP27^T13A^, TabZIP27^S24A^, TabZIP27^T52A^, TabZIP27^T13A/T52A^ protein was analyzed using phos-tag acrylamide. T: threonine; S: serine; A: alanine; substitution of Thr13 and/or Thr52 with alanine (A) removes the corresponding phosphorylation sites (T13A, T52A and T13A/T52A). (**B**) TaCIPK16-mediated TabZIP27 stability in cell-free degradation assays in the protein extracts of WT. T: threonine; D: aspartic acid. (**C**) The phenotypes of WT and *TaCIPK16*-overexpression wheat seedlings were grown with or without Cd for 14 d. Scale bar = 5 cm. (**D**) The dry weight of WT and *TaCIPK16* overexpression wheat seedlings were grown with or without Cd for 14 d. (**E**) The Cd content in root of WT and *TaCIPK16* overexpression wheat seedlings were grown with or without Cd for 14 d. (**F**) The Cd content in shoot of WT and *TaCIPK16* overexpression wheat seedlings were grown with or without Cd for 14 d. Data are means ± SD (n = 3) and different letters above bars indicate significant differences by one-way ANOVA followed by Tukey’s HSD test at *p* < 0.05. (**G**) Detection of protein abundance of TabZIP27 in the WT and *TaCIPK16* overexpression wheat. Total proteins of WT and *TaCIPK16* overexpression wheat were collected before Cd treatment and 72 h after treatment. (**H**) Phosphorylation status of TabZIP27 in *TaCIPK16* overexpression lines under normal and Cd conditions. Anti-actin as the loading control.

Given that phosphorylation modifications can influence protein stability, we next examined whether the phosphorylation of TabZIP27 mediated by TaCIPK16 affects its stability. Initially, we utilized online software to predict the phosphorylatable amino acids in TabZIP27, identifying Thr13, Ser24, and Thr52 as the three highest-scoring residues. To investigate the identified phosphorylation sites, we mutated Thr13, Ser24, and Thr52 to alanine (A), creating TabZIP27 mutants that are incapable of phosphorylation. Following this, we performed in vitro phosphorylation assays using recombinant proteins. The results indicated that the phosphorylation levels of both TabZIP27^T13A^ and TabZIP27^T52A^ were diminished compared to WT TabZIP27, thereby indicating that Thr13 and Thr52 are major candidate sites contributing to TaCIPK16-mediated phosphorylation of TabZIP27 (Figure 6A). Furthermore, cell-free degradation assays showed that the phosphomimetic TabZIP27 variants exhibited slower degradation than WT TabZIP27 (Figure 6B), suggesting that phosphorylation at these sites may enhance TabZIP27 stability.

*TaCIPK16*-overexpressing wheat was generated through *Agrobacterium*-mediated transformation. Based on the expression levels of *TaCIPK16*, L2 and L3 were selected for subsequent functional studies (Supplementary Figure S7). The results demonstrated that overexpression of *TaCIPK16* enhanced the growth condition of wheat under Cd stress (Figure 6C), increased biomass (Figure 6D), and concurrently reduced Cd accumulation (Figure 6E,F). To further analyze the effect of TaCIPK16 on the stability of TabZIP27 in plants, we generated polyclonal antibodies against TabZIP27. The results of western blot indicated that in wheat not exposed to Cd, the protein abundance of TabZIP27 remained unaffected. However, following Cd treatment, TabZIP27 protein was degraded in WT wheat, whereas the degradation was less pronounced in TaCIPK16-overexpressing wheat (Figure 6G).

To investigate whether Cd stress affects TabZIP27 phosphorylation, we examined the phosphorylation status of TabZIP27 in the roots of WT and *TaCIPK16*-overexpressing wheat plants under control and Cd-stress conditions. Phos-tag analysis revealed phosphorylated forms of TabZIP27 in both WT and *TaCIPK16*-overexpressing plants, with a stronger phosphorylation signal in the *TaCIPK16*-overexpressing lines. Moreover, Cd treatment further increased TabZIP27 phosphorylation in both genotypes, with a more pronounced increase in the *TaCIPK16*-overexpressing lines (Figure 6H). The conventional western blot reflects the steady-state abundance of TabZIP27 (Figure 6G), whereas the Phos-tag blot reflects its phosphorylation status (Figure 6H). The higher abundance of TabZIP27 in *TaCIPK16*-overexpressing plants under Cd stress was accompanied by a higher level of TabZIP27 phosphorylation. Together with the protein degradation assays described above, these results suggest that *TaCIPK16*-mediated phosphorylation is associated with enhanced stability of TabZIP27 under Cd stress.

## 3. Discussion

Previous studies have reported that *Arabidopsis MAN3* and *MAN7* are involved in regulating Cd stress responses [13,22]. However, the mechanisms by which *MAN* genes regulate Cd accumulation in wheat remain unclear. Through transcriptome analysis of wheat under Cd stress, we identified a gene homologous to *Arabidopsis MAN7*, designated *TaMAN7-4B*, which was found to be upregulated in response to Cd stress (Supplementary Figure S1). We subsequently demonstrated, at both physiological and molecular levels, that *TaMAN7-4B* is a key gene involved in regulating Cd accumulation in wheat. Notably, the suppression of *TaMAN7-4B* expression significantly increased the sensitivity of wheat to Cd stress, as evidenced by a markedly higher Cd content and a significant reduction in biomass in RNAi wheat plants under Cd stress (Figure 1A,B). The binding of Cd by root cell walls serves as a primary line of defense against Cd accumulation; thus, the composition of cell walls influences Cd translocation to the shoot. For example, an increase in hemicellulose content within cell walls can enhance Cd chelation, effectively preventing Cd transport to organelles [35]. Mannan, a common hemicellulose polysaccharide, is composed of mannose units. Studies have confirmed that mannose regulates plant uptake of Cd. In *Cornus officinalis* polysaccharides, the structural units composed of mannose, galactose, and arabinose, with a molar ratio of 6.75:12.26:14.19, may inhibit Cd transmembrane transport through cell wall chelation [36]. Conversely, mannose in *Spinacia oleracea* forms complexes with other monosaccharides that can directly scavenge free radicals, demonstrating significant protective effects against Cd-induced cytotoxicity in vitro experiments involving HEK293 and HCT116 cells [37]. In this study, suppression of *TaMAN7-4B* expression significantly reduced the mannose content in wheat roots and decreased the amount of Cd retained by the cell wall and hemicellulose. In contrast, *TaMAN7-4B* overexpression increased the mannose and hemicellulose contents and enhanced Cd retention in these cell wall fractions (Figure 3A–H). In contrast, overexpression of *TaMAN7-4B* produced entirely opposite results (Figure 3A–D). These findings suggest that *TaMAN7-4B*-mediated Cd tolerance in wheat is dependent on the mannose content in the cell wall.

Research has demonstrated that various families of TFs, including WRKY, MYB, bZIP, and NAC, play a crucial role in regulating plant responses to Cd stress. They achieve this by specifically binding to promoter elements of downstream target genes, which subsequently influences processes related to Cd uptake, transport, and detoxification. In maize, NAC TFs can produce intron-retained splicing variants in response to Cd stress. These variants inhibit the expression of target genes in a dominant-negative manner by forming heterodimers with their full-length NAC proteins, thereby impacting Cd tolerance [38]. Similarly, members of the bZIP family regulate the transcription of antioxidant enzymes and metal transporters under Cd stress by forming homo- or heterodimers that bind to DNA sequences containing the ACGT core [39]. Previous studies have indicated that *Arabidopsis* bZIP44 directly regulates the expression of *MAN7* [13]. In this study, we identified that wheat TabZIP27 binds to the promoter of *TaMAN7-4B* and regulates its expression (Figure 4), suggesting a similarity in the regulatory mechanisms governing *TaMAN7-4B* expression in wheat and *MAN7* expression in *Arabidopsis*.

Phosphorylation modification, one of the most prevalent post-translational modification mechanisms in eukaryotes, plays a pivotal role in regulating the stability and functional execution of plant TFs. Studies demonstrate that CIPK directly modulates the expression of stress-responsive genes, such as RD29A and COR15A, by phosphorylating members of TFs families including bZIP, WRKY, and MYB. This process enhances plant adaptability to drought, high salinity, and oxidative stress [1]. For instance, within the ABA signaling pathway, CIPK6 phosphorylates the ABF2/AREB1 TF, promoting its binding capacity to abscisic acid response elements (ABRE) and activating the expression of genes associated with the synthesis of osmoregulatory substances [40]. This precise post-translational modification mechanism not only prevents energy consumption associated with continuous activation but also facilitates the specific transmission of stress signals, reflecting the evolutionary advantage of plant signaling networks. To investigate whether the protein stability of TabZIP27 is influenced by phosphorylation modification, we identified the interacting proteins of TabZIP27 and found that TaCIPK16 is a potential interaction partner of TabZIP27 (Figure 5A–D). At the molecular level, the impact of phosphorylation modifications on the stability of TFs demonstrates bidirectional regulatory characteristics. On one hand, certain phosphorylation events can enhance the binding of TFs to E3 ubiquitin ligases, thereby accelerating their proteasomal degradation [41]. Conversely, as observed in members of the bZIP family, specific phosphorylation can stabilize protein conformation and shield ubiquitination sites, thus prolonging their half-life [42]. To investigate the effect of TaCIPK16-mediated phosphorylation on the stability of TabZIP27, we conducted cell-free degradation assays using wheat protein extracts to assess the stability of TabZIP27 and its mutants. The results indicated that the degradation rates of TabZIP27^T13D^, TabZIP27^T52D^, and TabZIP27^T13D/T52D^ were significantly slower, while degradation of TabZIP27 was pronounced. Additionally, MG132 effectively inhibited the degradation of TabZIP27, suggesting that the stability of TabZIP27 is regulated by the 26S proteasome pathway (Figure 6B). These findings imply that the 26S proteasome-mediated degradation of TabZIP27 is closely linked to its phosphorylation status.

Based on molecular biological evidence, we conclude that TaCIPK16 enhances the protein stability of TabZIP27 by interacting with and phosphorylating it, thereby promoting TabZIP27-mediated transcription of *TaMAN7-4B*. This process increases the binding of Cd to the wheat root cell wall and inhibits Cd translocation to the shoots. Therefore, our findings demonstrate the crucial molecular mechanism of the TaCIPK16-TabZIP27-*TaMAN7-4B* module in controlling Cd accumulation in wheat. This study provides deeper insights into the molecular network of wheat’s response to Cd stress and identifies feasible candidate genes for reducing Cd accumulation in wheat.

## 4. Materials and Methods

### 4.1. Plant Materials and Growth Conditions

Mutants exhibiting downregulated expression of *TaMAN7-4B* and *TaCIPK16* were developed in the Fielder background using the RNA interference (RNAi) system. To generate transgenic plants overexpressing *TaMAN7-4B* or *TaCIPK16*, the open reading frames (ORFs) of *TaMAN7-4B* and *TaCIPK16* were cloned into the pCAMBIA1301 vector under the control of the *35S* promoter. These constructs were subsequently introduced into the Fielder wheat line through *Agrobacterium tumefaciens*-mediated genetic transformation. The wheat plants were cultivated in a growth chamber under a photoperiod of 16 h of light and 8 h of darkness at a temperature of 24 °C and 60% relative humidity. The plants were grown in a half-strength Hoagland nutrient solution until reaching the three-leaf stage, at which point they were divided into control and treatment groups. The control group continued to receive half-strength Hoagland nutrient solution, while the treatment group was subjected to a nutrient solution containing 50 μM CdSO_4_.

### 4.2. RNA Extraction and Reverse Transcription Quantitative PCR (RT-qPCR)

Total RNA was isolated from various tissues using Trizol reagent (CwBio, Suzhou, China) according to the manufacturer’s instructions. Reverse transcription was performed with reverse transcriptase (CwBio, Suzhou, China), followed by RT-qPCR analysis using SYBR Green reagent (CwBio, Suzhou, China), with *TaActin* (LOC123179078) serving as the internal controls. The relative changes in gene expression were quantified based on three biological replicates, employing the 2^−ΔΔCt^ method [43].

### 4.3. Measurement of Chlorophyll Content

Chlorophyll a, chlorophyll b, and total chlorophyll contents were measured using a plant chlorophyll content assay kit (Solarbio, Beijing, China), following the manufacturer’s instructions.

### 4.4. Determination of Cd Content

After 14 days of Cd treatment, fresh roots and shoots of wheat were collected, washed with deionized water (ddH_2_O), and then dried in an oven at 70 °C for 48 h. The dried samples were weighed and digested with 4 mL of HNO_3_/HClO_4_ (4:1, *v*/*v*) at 130 °C until the solution became clear. All samples were then diluted to 10 mL with deionized water, filtered, and stored for further analysis. The Cd concentration was determined using Inductively coupled plasma optical emission spectroscopy (ICP-OES, iCAP 7000, Thermo Fisher Scientific, Waltham, MA, USA).

### 4.5. Extraction of Cell Wall and Hemicellulose

The extraction of cell walls and hemicellulose was conducted following the protocol established by Wu et al. [26]. Initially, the roots were harvested and ground into a fine powder using liquid nitrogen. This powder was then subjected to shaking in 75% (*v*/*v*) ethanol for 20 min, followed by centrifugation at 9000 rpm for 10 min. The supernatant was discarded, and the resulting pellet was resuspended and washed with acetone for another 20 min. After a second centrifugation at 9000 rpm for 10 min, the supernatant was discarded once more. Subsequently, the pellet was washed with a methanol:chloroform mixture (1:1, *v*/*v*) for 20 min, followed by another centrifugation at 9000 rpm for 10 min, after which the supernatant was removed. Finally, the pellet underwent a wash with methanol for 20 min, was centrifuged at 9000 rpm for 10 min, and the supernatant was discarded. The resultant pellet constituted the cell wall fraction. Pectin was extracted from the previously mentioned cell wall preparation. Approximately 2 mg of cell wall material was incubated in 1 mL of sterile water at 100 °C for one hour. Following centrifugation at 15,000 rpm for 20 min, the supernatant was collected, and this process was repeated twice. The supernatants obtained from these three rounds were combined to form the pectin fraction. Hemicellulose was extracted from the remaining pellet after the pectin extraction. Subsequently, 1 mL of 24% (*w*/*v*) KOH and 0.02% (*w*/*v*) KBH_4_ were added to the pectin precipitate and dissolved at room temperature for 12 h. After centrifugation at 15,000 rpm for 10 min, the supernatant was transferred into a 5 mL tube. The aforementioned steps were repeated, and the total supernatant was designated as the hemicellulose fraction.

To determine the Cd content in the cell wall, approximately 2 mg of cell wall powder was co-incubated with 1 mL of 2 mM HCl and incubated for 24 h under shaking conditions. The Cd concentration in the extract was measured using ICP-AES (iCAP 7000, Thermo Fisher Scientific, Waltham, MA, USA) after dilution. For the determination of Cd in hemicellulose, the supernatant containing the extracted hemicellulose was diluted and analyzed similarly by ICP-AES (iCAP 7000, Thermo Fisher Scientific, Waltham, MA, USA).

### 4.6. Yeast One-Hybrid Assay

Yeast one-hybrid assays were carried out using the Matchmaker Yeast One-Hybrid System (Clontech). The promoter region of *TaMAN7-4B* was cloned into the pAbAi vector and subsequently integrated into the Y1H Gold yeast genome after restriction enzyme digestion. The coding sequence (CDS) of *TabZIP27* was cloned into the pGADT7 (AD) vector. The resulting plasmids, pGADT7-*TabZIP27* and pAbAi-*TaMAN7*-*4Bprom*, were transformed into Y1H Gold yeast cells and cultured at 30 °C for three days on SD medium lacking uracil (SD-Ura), under two conditions: with or without the addition of 200 ng/mL Aureobasidin A (AbA200). The combination of pAbAi and pGADT7-*TabZIP27* plasmids served as the negative control.

### 4.7. Electrophoretic Mobility Shift Assay (EMSA)

The EMSA was conducted using a chemiluminescent EMSA kit (Beyotime, Shanghai, China) following the manufacturer’s instructions. Biotin-labeled DNA probes corresponding to the promoter region of *TaMAN7-4B* were synthesized, with the biotin moiety attached to the 5′end of the probes. Unlabeled DNA fragments, either matching the sequence or containing mutations, served as competitors. The recombinant TabZIP27-GST protein was utilized in the binding reaction to assess its binding affinity to the probes.

### 4.8. Yeast Two-Hybrid Assay

The yeast library screening was conducted using the Matchmaker GAL4 Gold Two-Hybrid System 3 (Clontech). The Y2HGold yeast strain containing *TabZIP27*-pGBKT7 (BD) was mated with the Y187 yeast strain, which harbored a wheat cDNA library constructed in pGADT7 (AD). The screening was performed on synthetic defined (SD) medium lacking adenine, histidine, leucine, and tryptophan (-Ade/-His/-Leu/-Trp). To further validate the interaction in yeast, *TabZIP27*-pGBKT7 and *TaCIPK16*-pGADT7 were co-transformed into the Y2HGold yeast strain. The transformants were subsequently cultured on SD medium lacking leucine and tryptophan (SD/-Leu/-Trp) and on SD medium lacking adenine, histidine, leucine, and tryptophan (SD/-Ade/-His/-Leu/-Trp) for three days.

### 4.9. GST Pull-Down Assay

The GST pull-down assay was conducted following the protocol described by Sun et al. [44]. The ORF of *TabZIP27* was cloned into the pGEX-4T-1 vector, while *TaCIPK16* was inserted into the pET-28a vector. The resulting recombinant plasmids were individually transformed into *Escherichia coli* BL21 (DE3) to express the recombinant proteins GST-TabZIP27 and His-TaCIPK16. Initially, the purified GST-TabZIP27 or GST was incubated with glutathione resin beads (Yeasen) at 4 °C for 3 h, followed by a 2-h incubation with His-TaCIPK16. After elution, the proteins were analyzed via immunoblotting using anti-His antibody (Abcam, 15149) and anti-GST antibody (Abcam, 9085), respectively.

### 4.10. Bimolecular Fluorescence Complementation (BiFC) Assay

In this study, the BiFC assay utilized the vectors pBiFC-VC155 and pBiFC-VN155. The open ORF of *TabZIP27* was cloned into pBiFC-VC155, while *TaCIPK16* was cloned into pBiFC-VN155. Subsequently, the recombinant plasmids *TabZIP27*-cYFP and *TaCIPK16*-nYFP were transiently co-expressed in the leaves of 4-week-old tobacco (*Nicotiana benthamiana*) using an *Agrobacterium*-mediated expression system. After a 48-h incubation period, YFP fluorescence was detected using confocal laser scanning microscopy (Olympus FV500, Olympus Corporation, Tokyo, Japan).

### 4.11. Co-Immunoprecipitation (Co-IP) Analysis

*TabZIP27* and *TaCIPK16* were cloned into the vectors pCambia1301-GFP and pCambia1307-MYC, respectively, resulting in the recombinant constructs TabZIP27-GFP and TaCIPK16-MYC. These two constructs were then infiltrated separately or in combination into tobacco leaves. After 48 h, total proteins were extracted in lysis buffer [50 mM Tris-HCl (pH 7.5), 150 mM NaCl, 1 mM EDTA, 1% (*v*/*v*) Triton X-100, 10% (*v*/*v*) glycerol, 1 mM dithiothreitol, 1 mM phenylmethylsulfonyl fluoride, and a protease inhibitor cocktail]. The extracts were pre-cleared with protein A/G magnetic beads (Beyotime) at 4 °C for 1 h to eliminate non-specific binding, and the supernatants were then incubated with the beads at 4 °C for 4 h with gentle rotation. The immunoprecipitates were washed three times with lysis buffer and eluted in SDS-PAGE loading buffer, followed by western blot analysis using anti-GFP (Abcam, ab290) or anti-MYC (Abcam, ab56) antibodies. Three independent biological replicates were performed.

### 4.12. Dual Luciferase Reporter Assay

The 2000 bp *TaMAN7-4B* promoter sequence was cloned into the pGREEN II 0800-LUC vector to construct a reporter gene, with the Renilla luciferase (REN) gene driven by the 35S promoter serving as the internal reference. The ORF of *TabZIP27* was cloned into the pGREENII 62-SK vector to create the effector gene. Both constructs were transiently co-transformed into tobacco using the *Agrobacterium*-mediated transformation system. Following an incubation period of 48 to 72 h, the activities of LUC and REN were measured using the Dual-Luciferase Reporter Assay Kit (Beyotime).

### 4.13. Phosphorylation Assay

In vitro phosphorylation was detected using the phos-tag kit (Wako Pure Chemical Industries, Ltd., Osaka, Japan). Purified TabZIP27-GST and TaCIPK16-His proteins were incubated at a 2:1 ratio in phosphorylation reaction buffer, which consisted of 20 mM Tris-HCl (pH 7.4), 30 mM MgCl_2_, and 1 mM DTT, along with 0.5 mM ATP. The reaction was incubated at 30 °C for 30 min. The phosphorylated proteins were subsequently separated by SDS-PAGE employing 20 μM phos-tag and 40 μM MnCl_2_. Western blot analysis was performed using a GST antibody. The experimental and control groups were supplemented with or without the calf intestine alkaline phosphatase (CIAP) respectively. The phosphorylation sites of TabZIP27 were predicted using the PPSP website (http://ppsp.biocuckoo.org/, accessed on 12 May 2025). Candidate phosphorylated amino acids were mutated to non-phosphorylatable alanine (A) through site-directed mutagenesis. Following this protocol, in vitro phosphorylation assays were executed to identify the amino acids in TabZIP27 that were phosphorylated by TaCIPK16.

To analyze protein phosphorylation in plants, the recombinant plasmids GFP-TaCIPK16 and MYC-TabZIP27 were transiently expressed in tobacco leaves using Agrobacterium-mediated transformation. After a 48-h incubation period, total proteins were extracted. Subsequently, Western blotting was conducted to detect phosphorylated proteins using anti-GFP and anti-MYC (Abcam, ab56).

### 4.14. Cell-Free Protein Degradation Assay

The cell-free degradation assay was conducted as previously described [45]. Briefly, total proteins were extracted from wheat seedlings at the three-leaf stage using an extraction buffer composed of 25 mM Tris--HCl (pH 7.5), 10 mM NaCl, 10 mM MgCl_2_, 5 mM DTT, and 1 mM phenylmethylsulfonyl fluoride. The phosphorylation sites were mutated individually into aspartic acid (D) to mimic phosphorylation. The purified proteins GST-TabZIP27, GST-TabZIP27^T13D^, GST-TabZIP27^T52D^, and GST-TabZIP27^T13D/T52D^ were incubated with the extracted soluble total protein in a reaction buffer containing 25 mM Tris-HCl (pH 7.5), 10 mM NaCl, 10 mM MgCl_2_, 4 mM PMSF, 5 mM DTT, and 10 mM ATP for 60 min. Subsequently, the amount of GST-TabZIP27 protein was detected using western blot analysis with a GST antibody.

### 4.15. Protein Degradation Assay In Vivo

The recombinant vectors GFP-TabZIP27, GFP-TabZIP27^T13D^, GFP-TabZIP27^T52D^, and GFP-TabZIP27^T13D/T52D^, along with Myc-TaCIPK16, were constructed and transformed into the *Agrobacterium* strain GV3101. The transformed *Agrobacterium* was utilized for the transient infection of tobacco leaves. Following infiltration, the tobacco plants were initially cultured in darkness for two days, after which they were treated with 50 μM Cd for 72 h. Finally, the leaves were harvested for protein extraction. The amount of GFP-TabZIP27 protein was subsequently analyzed using western blotting with an anti-GFP antibody.

### 4.16. Statistical Analysis

All experiments were performed with at least three independent biological replicates (n = 3). Significant differences between two groups were determined using a two-tailed Student’s *t*-test. For comparisons among three or more groups, a one-way analysis of variance (ANOVA) followed by Tukey’s honestly significant difference (HSD) test was used for pairwise multiple comparisons. A *p* value < 0.05 was regarded as statistically significant, and data are presented as means ± standard deviation (SD).

### 4.17. Primers

The primers are shown in Supplementary Table S1.

## Data Availability

The data supporting the findings of this study are available from the corresponding author upon reasonable request.

## Supplementary Materials

The following supporting information can be downloaded at: https://www.mdpi.com/article/10.3390/plants15203214/s1, Figure S1: RT-qPCR analysis of the expression *TaMAN7-4B* under Cd treatment at different time points (hour). Figure S2: RT-qPCR analysis of the expression *TaMAN7-4B* in *TaMAN7-4B* RNAi wheat. Figure S3: RT-qPCR analysis of the expression *TaMAN7-4B* in *TaMAN7-4B* overexpression wheat. Figure S4: Cd transfer coefficient in WT, *TaMAN7-4B* overexpression, and *TaMAN7-4B* RNAi wheat. Figure S5: Promoter sequence of *TaMAN7-4B*. Figure S6: (A) Subcellular localization of TabZIP27. (B) Subcellular localization of TaCIPK16. Figure S7: RT-qPCR analysis of the expression *TaCIPK16* in *TaCIPK16* overexpression wheat. Table S1: Primers and probes used in this study.
