# Supplementary material for: Phosphorylation of TabZIP27 by TaCIPK16 Regulates TaMAN7-4B Expression During Cadmium Response in Wheat
Source: Plants (Basel). 2026 Oct 20;15(20):3214. doi: 10.3390/plants15203214 (PMC13709076; doi:10.3390/plants15203214)
Supplement: Supplementary file 1 [file plants-15-03214-s001.zip › Supplementary Figure.pdf]

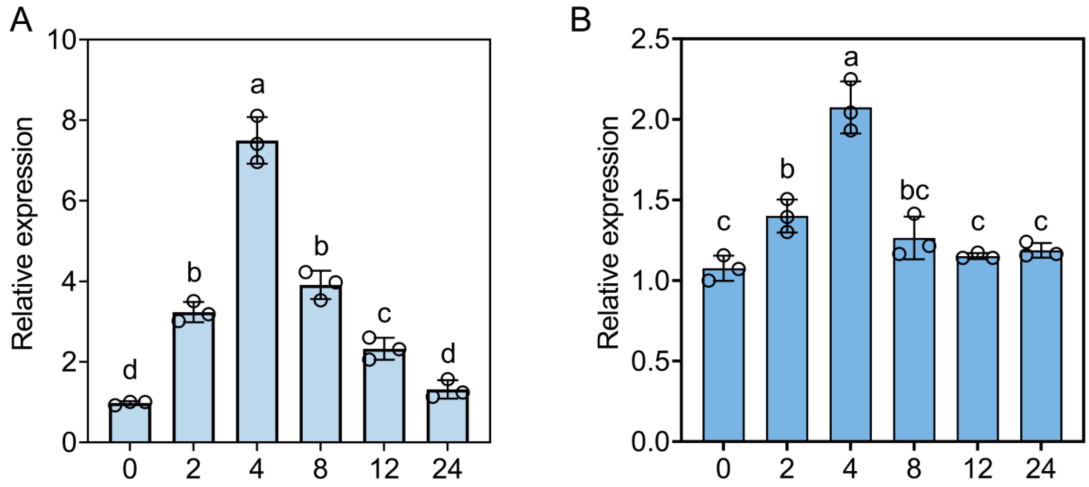

Supplementary Figure S1 RT-qPCR analysis of the expression *TaMAN7-4B* under Cd treatment at different time points (hour). (A) root tissue; (B) shoot tissue. Different lowercase letters indicate significant differences, $P < 0.05$. The data are from three independent samples.

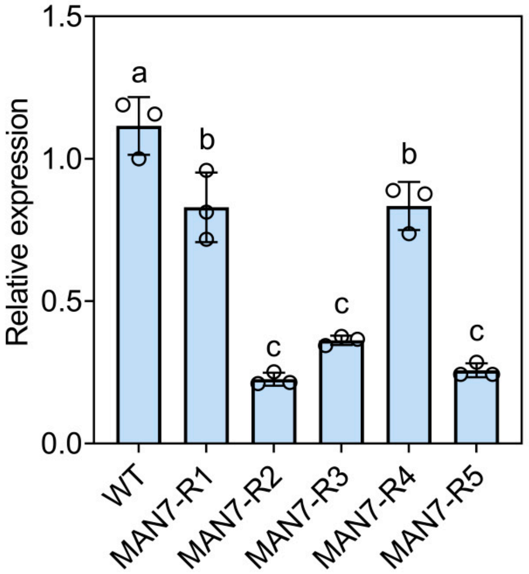

Supplementary Figure S2 RT-qPCR analysis of the expression *TaMAN7-4B* in *TaMAN7-4B* RNAi wheat. Different lowercase letters indicate significant differences, $P < 0.05$. The data are from three independent samples.

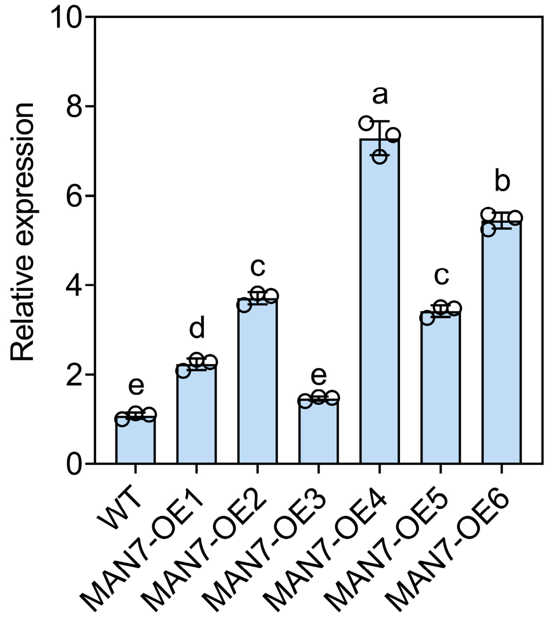

Supplementary Figure S3 RT-qPCR analysis of the expression *TaMAN7-4B* in *TaMAN7-4B* overexpression wheat. Different lowercase letters indicate significant differences, $P < 0.05$. The data are from three independent samples.

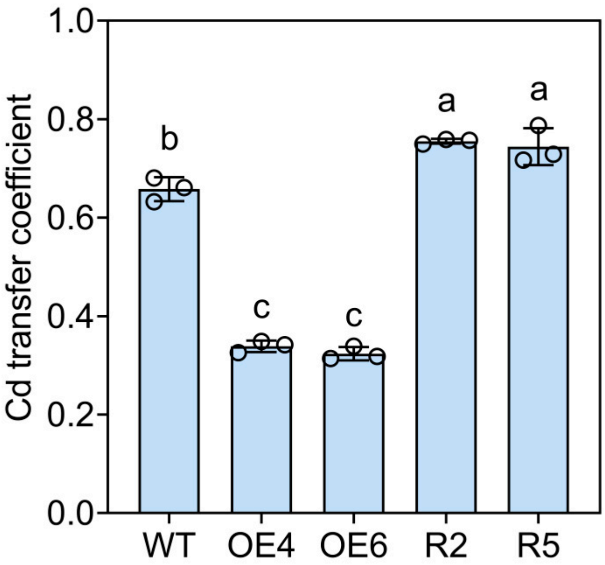

Supplementary Figure S4 Cd transfer coefficient in WT, *TaMAN7-4B* overexpression, and *TaMAN7-4B* RNAi wheat. Different lowercase letters indicate significant differences, $P < 0.05$. The data are from three independent samples.

TTCTCGAAAATACCTCTTCCTTCCTTGAGAGCCGAGGAACAAACTAAGTTTCAGGAA
GGCACTCATGATTGCTCCTCAAATGGCGTTGCAGATAGGGTGCCTGTCTTTATCAGAA
TCGCACCTTCAAACACTGAAATGGAGCGATATGCAGTTATGCACATACAGGGTAGCA
GGAGTCGATCAGGGCCACATACTATTACTAGAAGAGATCACACGCGAGTCGCTACAG
ACCAATTGCAGCTATTCTTTTCTTTTTCTACCTTCACGCAGTGAGCGACGCGAGCGTG
GCGGGGCAGTTCATCTTCATCGCGACCAACCGTACATTACTAGCTCAAGGACAAGAG
TGAAGCACGTGGGATTAGTTTACCGGTGGACTTTTTTTTCGAAACGAAGGTAAAAAGA
TTTGGTTAACGGAAAACCAGGCAAAAACCGTCACAATCCGTTGAGCGGTACACATAG
TATACCCCACACACTCCTTTCTAAAGAGGGCTTCGTCGAACTAGCACACATGCGTCAT
TAATCCCGAGCAAGCGACTCTAACTTGATCATAAACAAGTATCAACCCAACCCATTCC
ATAAGGGTCATGTGAAGATAAATGAAGATTTGCACCATTACTCAGCCACTCGCGTCCA
GGACGACGCAACACCGGCTCCGATGGAGAGCTACGAAGAACTATGTAGCGGCTGGT
GTGCCAGTAGGGTGAGTTGGCAACAAGGCTTCTATGGCAACGATGGCGGTGGTGAG
CGAGGCCGGAAACACGACAATGGTTGTATTAGTCGGCTCTTTTTTTTGTGTTTGTTGG
ATTGCTTTGGTTTGTTTGTTGCTGTGAAATCGGAGCTGCGGCGGCGGGCCCCCGTG
GTAACGATGACGAGCAGCAGGTGGTCTATTCGGTGGTCTTTATGGCGGGGGGGCCC
TCCAACTGACTCCCCCCTCTCCTTTCAAAGATGAAGCCCTCAAGAACGGCATGACGC
AGTATGCCGTCATCATCCGATCCATGAATGCACAGATCCGAGGTTTCCCTTGAAGAAA
GGGTCGGTATAGAGATGAGCGCTTCACCACGGCAACACCTTCAAAAAGATTGCGAC
GCCCACAAACGCCACCGTCGCGGCTTCGGCCATAACCGGAGTCAAGCTTTCGCCCC
ATGTCGTCGCACCACACCCATACCTAACCAACCGAACCTCCAGCAATGCATCACCAT
CTGTCGACGGAGAACAGCAAGGAGCCGCCTAGTCAGCCGCACTGTGCGGAGGCCG
CAAGCGTCCAGCCATCAAATCCAGCCGGCAAACCACGACTGGCCATATCCAAGACTT
CCCACCGCGGACTGAAGCCATCGCGCCCTGCCAGCACCGTGATGACATAATGTTCG
AAAATTCTAAAAAAAATGCATACTGGCAAAGGTATGTCCACGGCACAAAGTTTTGTGT
AAAAAAATATTTTTGTTTTGTCTCAGTAAAAAAGATAAATTTTACTGCTTCTAAATAGCG
TTTCACGGCATAAAATTTTGTCTTTTTTGCACAGGCTACAAAAAAAAGTTGTTTTTCTG
TGAAACTTTCTGCACACACATAGAATATGGAGATGTCCGCACGCATTTTTTTTTAAATT
TTAACATTGCAAAATGTGTTTTTTAAAATGAGTTCATATATATCCGGGTTCATCCATGCA
CTTCTCGGCTTGAGATGGATTTGGTAAACCCTACCGAGGACCGCAAAACCGTACTGT
TCTACCATCGTGTCCTTTTCACTACCCCATGTGAGGTGCCAATAAATTGCTTCCATGC
GCCGGTACCGACGAAGTTAATGTCCCCCTCTCCACCCCCACCCGCGTAACGAACCC
AAACTGTAGCTCAAAAAACAGCTCTGTGCACAAAATGGAACACCAAAGACCCCCTGA
CAAGTGGGTCTCCATCGTCTACAGTCTGAGATTTACTCAATGGCGCGACTGTGAAAC
GAAGCCTTGTAAGCTCTGGTCTATTTAAGCACCCACTGAGCTTCAGCTCACCG(-1)

Supplementary Figure S5 Promoter sequence of *TaMAN7-4B*. Core sequence targeted by TabZIP27 has been marked in red.

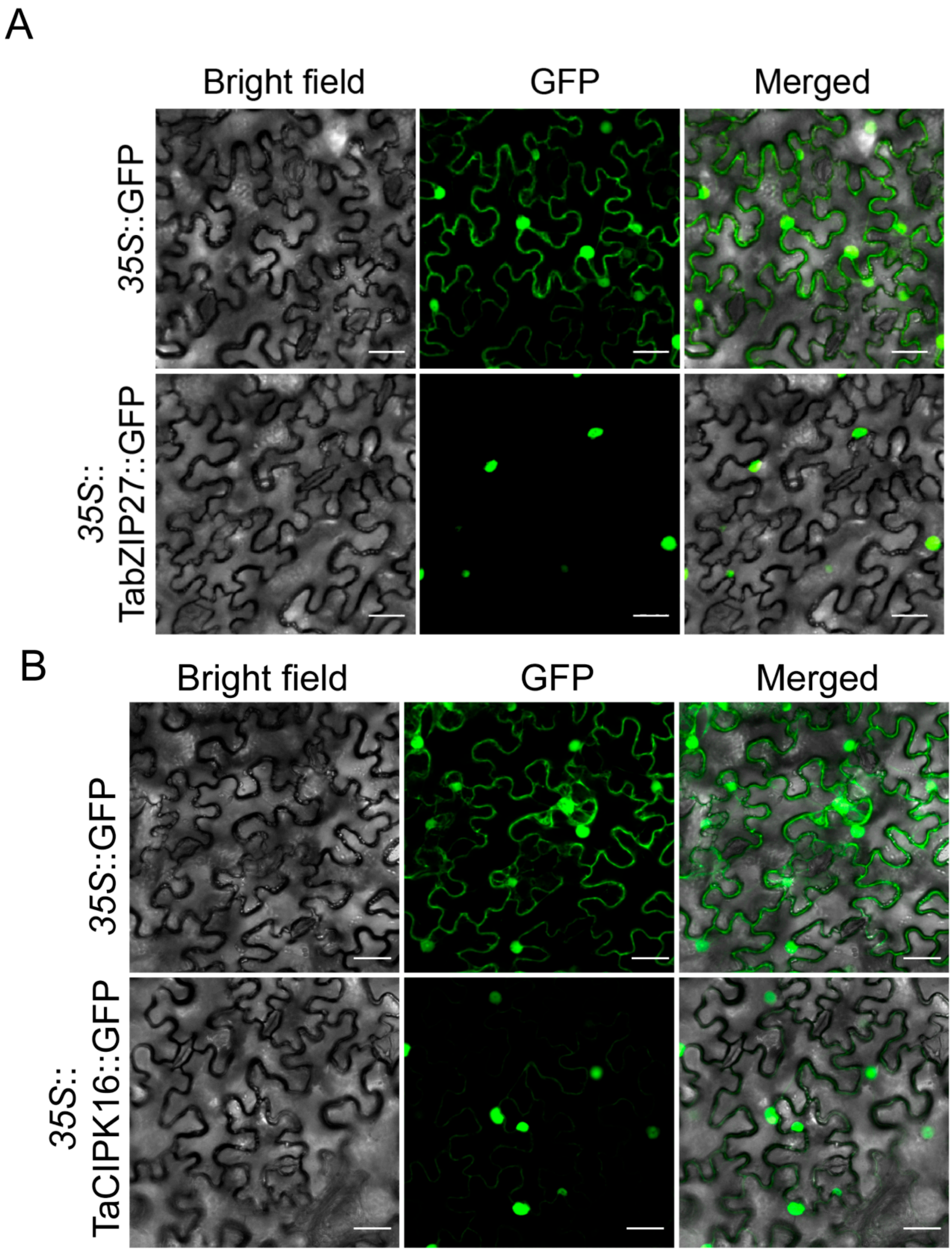

Supplementary Figure S6 (A) Subcellular localization of TabZIP27. (B) Subcellular localization of TaCIPK16. Scale bar = 20 μm.

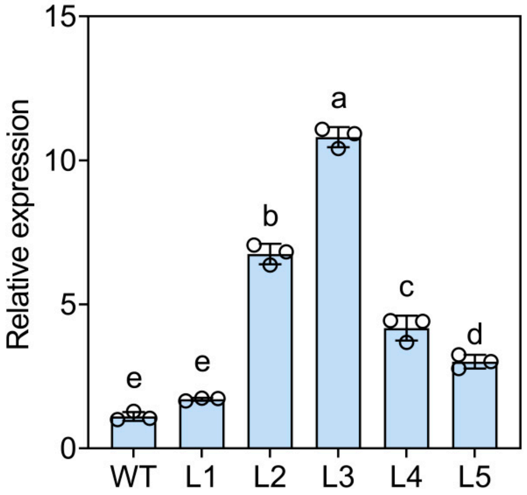

Supplementary Figure S7 RT-qPCR analysis of the expression *TaCIPK16* in *TaCIPK16* overexpression wheat. Different lowercase letters indicate significant differences, $P < 0.05$. The data are from three independent samples.
